# Decision making method for operational performance of Chinese commercial banks based on scenario fuzzy sets

**DOI:** 10.1038/s41598-025-04584-2

**Published:** 2025-06-05

**Authors:** Yingming Yang, Meilan Yang, Zhenpei Shan

**Affiliations:** Liupanshui Normal University, Liupanshui, 553004 China

**Keywords:** Improved scenario fuzzy set, Hamming distance measurement, Relative closeness, TOPSIS, Multi-attribute decision-making, Applied mathematics, Statistics

## Abstract

In the original fuzzy set, after optimization, we expanded it into four categories: positive, neutral, negative, and invalid, and applied them to the scenario fuzzy set. This improvement not only fills the gap in the application scope of scenario fuzzy sets in existing literature, but also provides a more comprehensive analytical framework for performance evaluation of commercial banks. Although there have been studies focusing on the classification and application of fuzzy sets, there is still a lack of in-depth exploration on the specific improvement and multidimensional evaluation of situational fuzzy sets. To this end, we propose an improved definition of scenario fuzzy sets and derive the Hamming distance. This distance provides a more comprehensive calculation method when dealing with multi-attribute decision-making problems, enabling decision-makers to more accurately evaluate the relationships between different attributes. By combining the Hamming distance measure and TOPSIS principle, we calculate the optimal value of relative closeness. By comparing the situation fuzzy set with the improved situation fuzzy set, the results show that the improved situation fuzzy set is superior to traditional methods in terms of comprehensiveness and accuracy. This study not only enhances the practical effectiveness of scene fuzzy sets, but also provides a new perspective for subsequent academic research.

## Introduction

Financial performance itself is complex and uncertain, which poses many challenges to the comprehensive performance evaluation of employees. Fuzzy logic provides an effective framework for addressing the common ambiguity and imprecision in performance evaluation, thereby making the analysis of various performance indicators more in-depth and detailed. Combining fuzzy sets with Multi Criteria Decision Making (MCDM) techniques enables decision-makers to comprehensively consider multiple conflicting factors such as profitability, risk management, and customer satisfaction to evaluate the performance of banks.

At present, research on performance evaluation of Chinese commercial banks mainly focuses on the relationship between borrowing and lending^1–3^, while comprehensive analysis of intermediary business is still insufficient, which leads to incomplete evaluation results^4^. In terms of loans^5,6^, Chinese commercial banks mainly rely on observable assets of enterprises, but it is often difficult to grasp those invisible assets that cannot be directly observed^7,8^. Usually, staff conduct indirect evaluations through conversations with the company^9,10^. Converting linguistic variables into fuzzy numbers can facilitate a more intuitive decision-making process, allowing decision-makers to express their evaluations using linguistic variables and convert them into fuzzy numbers ^11,12^. However, related performance is often difficult to reflect in compensation^13^^.^

Starting from the operational performance scenario of Chinese commercial banks^14^, an analysis was conducted on the performance salary evaluation indicators of deposits, intermediary business, and loans in the operating departments^15^. The performance ratios were (0.55, 0.05, 0.40), and departmental performance mainly relied on deposits and loans, while intermediary business accounted for a relatively small proportion^1^. Therefore, many Chinese commercial banks did not carry out intermediary business, leading to customer loss in Chinese commercial banks. If a certain Chinese commercial bank wants to conduct performance evaluations for outstanding employees at the end of the year, they will quantify their deposits and loans separately. Employees A and B have deposits and loans of (0.50, 0.40), which cannot be evaluated as excellent. Therefore, the Chinese commercial bank will also include intermediary business in the assessment scope, and employees A and B will quantify their deposits and loans of (0.50, 0, 0.40), and they will carry out intermediary business^16^. However, it was not effective, so it was still not possible to evaluate excellence. Therefore, the evaluation can only be based on whether the two individuals have conducted business or not, i.e. (0.50, 0, 0.40, $\zeta$). The more intermediate business they have conducted under the same conditions, the better. However, intermediary business plays a crucial role in the sustainable development of Chinese commercial banks. It is not scientific to measure the quality of Chinese commercial banks solely based on their deposits and loans. It is more reasonable to include intermediary business in departmental performance evaluation of Chinese commercial banks.

Some scholars have applied directed measures (RDM) and cross efficiency models^17^ to the multi period and multi-stage evaluation of banks’ fixed assets, deposits, and non-performing loans of enterprises. Meanwhile, intuitionistic fuzzy numbers ^18^ and data envelopment analysis (DEA) models have also been used to evaluate the performance of Chinese commercial banks^14,19–22^. Due to the uncertainty of employee performance, it is difficult to accurately calculate their average value. Therefore, the Fuzzy Analytic Hierarchy Process (FAHP) combined with the Similarity Ideal Solution (TOPSIS)^23^ method is proposed for performance ranking, and the proposed evaluation model is integrated to evaluate the performance of bank employees. The multiple evaluation attributes of multi-attribute decision-making (MADM) problems have both qualitative and quantitative properties^24–26^, which makes employee performance evaluation face certain difficulties. Therefore, a ranking model combining Fuzzy Analytic Hierarchy Process (FAHP) and Similarity Ideal Solution (TOPSIS) method has emerged^27^. In addition, the slack measurement (SBM) method under the data envelopment analysis (DEA) model is widely used to evaluate bank efficiency. This method not only improves the accuracy of efficiency estimation, but also enables detailed analysis of different bank types^28^. In addition, the sustainable development of banks plays an important role in promoting high-quality economic development in China^29^. However, the operational performance of Chinese commercial banks has ambiguity in the evaluation process, making fuzzy sets more persuasive in assessing the operational performance of Chinese commercial banks. Reducing risks is particularly important in the rapidly changing financial environment^30,31^.

Although there have been numerous studies exploring the rationality and effectiveness of performance indicators in the field of commercial bank performance evaluation, significant ambiguity still exists in the operational performance evaluation process of Chinese commercial banks. Current literature mostly focuses on traditional performance evaluation models, neglecting how to effectively reduce risks and cope with uncertainty in the rapidly changing financial environment ^26,27^. Therefore, the innovation of this study lies in proposing an improved scenario fuzzy set method based on fuzzy set theory, specifically for evaluating the operational performance of Chinese commercial banks^28–30,32^. Firstly, the concept of improved scenario fuzzy set is introduced^14^, which can comprehensively and flexibly evaluate performance indicators, helping banks better cope with complex market environments. Secondly, based on the principle of the Hamming distance measurement formula^33^, an improved Hamming distance measurement formula is constructed and combined with TOPSIS theory to define relative closeness and improve the accuracy and flexibility of evaluation. This method provides higher accuracy and flexibility compared to the existing research gaps in fuzziness assessment.

## Preliminaries

This section introduces the basic concepts of situational fuzzy sets to support subsequent research efforts.

### Scenario fuzzy set

#### Definition 1.1^34^

Let $X$ be a domain of discourse, and the fuzzy set on $X$ is defined as $A = \{ (x,u_{A} (x))|x \in X\} u_{A} (x)$ representing the membership degree of element $x$ to set $X$.

#### Definition 1.2^35^

Let $X$ be a domain of discourse, where the intuitionistic fuzzy set on $X$ is $A = \{ (x,u_{A} (x),v_{A} (x))|x \in X\}$ $u_{A} (x),{\text{v}}_{A} (x)$, $x$ has membership and non membership degrees with respect to set $A$, and $u_{A} :x \to [0,1]$ and $v_{A} :X \to [0,1][0,1]$; There is $x \in X,0 \le u_{A} (x) + v_{A} (x) \le 1$.

#### Definition 1.3^34^

Let $X$ be a domain of discourse, with respect to the hesitation $\pi_{A} (x) = 1 - u_{A} (x) - v_{A} (x)$ $x \in X,0 \le \pi_{A} (x) \le 1$ of set A.

Scenario Fuzzy Set (PFS) is an extension of Intuitive Fuzzy Set, which plays a decisive role in describing uncertain information to solve practical problems. Its definition is as follows:

#### Definition 1.4

Let $X$ be a domain of discourse, and define.

$$A = \{ (x,u_{A} (x),\eta_{A} (x),v_{A} (x))|x \in X\}$$, $$u_{A} (x) \in [0,1]$$.

as a scenario fuzzy set A on $X$, representing the positive membership degree of $x$ with respect to the set $A$, $\eta_{A} (x) \in [0,1]$ as the neutral membership degree of $x$ with respect to the set $A$, $v_{A} (x) \in [0,1]$ as the negative membership degree of $x$ with respect to the set $A$, and for $x \in X$, $u_{A} (x){ + }\eta_{A} (x){ + }v_{A} (x) \le 1$.

#### Definition 1.5

Setting the rejection membership degree of $x$ with respect to set $A$ as $\xi_{A} (x) = 1 - u_{A} (x) - \eta_{A} (x) - v_{A} (x)$ and $\alpha = (u_{A} (x),\eta_{A} (x),(v_{A} (x))$ is called the situational fuzzy element or situational fuzzy value $u_{A} (x) \in [0,1],\eta_{A} (x) \in [0,1],v_{A} (x) \in [0,1]$, when $\xi_{A} (x) = 0$ is an intuitionistic fuzzy set.

#### Definition 1.6

Let $\alpha = (u_{A} (x),\eta_{A} (x),v_{A} (x))$ be a scenario fuzzy element. The exact function of scene ambiguity is:

1 $$H(\alpha ) = u_{A} (x){ + }\eta_{A} (x){ + }v_{A} (x)$$

Scoring function:

2 $$S(\alpha ) = u_{A} (x) - v_{A} (x)$$

of which: $$H(\alpha ) \in [0,1],S(\alpha ) \in [0,1]$$.

#### Definition 1.7

Let $X = \{ x_{1} ,x_{2} , \ldots ,x_{n} ,\}$ be a domain of discourse, and $Q_{1}$ and $Q_{2}$ be scenario fuzzy sets on $X$, Then Haiming distance

3 $$d(Q_{1} ,Q_{2} ) = \frac{1}{3n}\sum\limits_{i = 1}^{n} {\left[ {\left| {u_{{Q_{1} }} (x_{i} ) - u_{{Q_{2} }} (x_{i} )} \right| + \left| {\eta_{{Q_{1} }} (x_{i} ) - \eta_{{Q_{2} }} (x_{i} )} \right| + \left| {v_{{Q_{1} }} (x_{i} ) - v_{{Q_{2} }} (x_{i} )} \right|} \right]}$$

#### Definition 1.8

Let $Q_{1}$ and $Q_{2}$ be a scenario fuzzy set of the domain $X = \{ x_{1} ,x_{2} , \ldots ,x_{n} \}$, where $W = \{ \omega_{1} ,\omega_{2} , \cdots ,\omega_{n} \}$ is the weight of the element, and $\omega_{{\text{i}}} \in [0,1](i = 1,2, \cdots ,n),\sum\limits_{i = 1}^{n} {\omega_{i} = 1}$ is the weighted Hamming distance between $Q_{1}$ and $Q_{2}$, which is.

4 $$d_{p} (Q_{1} ,Q_{2} ) = \frac{1}{3n}\sum\limits_{i = 1}^{n} {\omega_{i} \left\{ {\left[ {\left| {u_{{Q_{1} }} (x_{i} ) - u_{{Q_{2} }} (x_{i} )} \right| + \left| {\eta_{{Q_{1} }} (x_{i} ) - \eta_{{Q_{2} }} (x_{i} )} \right| + \left| {v_{{Q_{1} }} (x_{i} ) - v_{{Q_{2} }} (x_{i} )} \right|} \right]} \right\}}$$

### Improvement of situational fuzzy sets(ISFS)

Due to the limitations of situational fuzzy sets, they are not conducive to analyzing practical problems. Therefore, improvements are made to the original situational fuzzy sets to better apply distance measures on them.

#### Definition 2.1

Let X be a domain of discourse and an improved situational fuzzy set $A$ on X, $A = \{ (x,u_{A} (x),\eta_{A} (x),v_{A} (x),\theta_{A} (x))|x \in X\}$, Among them, $u_{A} (x) \in [0,1]$ represents the positive membership degree of the $x$ set $A$, represents the neutral membership degree of the set, $\eta_{A} (x) \in [0,1]$ represents the negative membership degree of the $x$ set $A$, $\theta_{A} (x) \in [0,1]$ represents the invalid membership degree of the $x$ set $A$, and for $x \in X$, $u_{A} (x){ + }\eta_{A} (x){ + }v_{A} (x){ + }\theta_{A} \le 1$. Then reject the membership degree as $\rho_{A} (x) = 1 - u_{A} (x) - \eta_{A} (x) - v_{A} (x) - \theta_{A}$, and call $\alpha = (u_{\alpha } ,\eta_{\alpha } ,v_{\alpha } ,\theta_{\alpha } )$ an improved scenario fuzzy element, and $u_{\alpha } \in [0,1],\eta_{\alpha } \in [0,1],v_{\alpha } \in [0,1],\theta_{\alpha } \in [0,1]$, $u_{\alpha } { + }\eta_{\alpha } { + }v_{\alpha } { + }\theta_{\alpha } \le 1$, when $\rho_{A} (x) = 0$ is the original scenario fuzzy set.

#### Definition 2.2

Let $\alpha = (u_{\alpha } ,\eta_{\alpha } ,v_{\alpha } ,\theta_{\alpha } )$ be an improved scenario fuzzy element, and its function $f(\alpha )$ be represented as $f(\alpha ) = u_{\alpha } { + }\eta_{\alpha } { + }v_{\alpha } { + }\theta_{\alpha }$, $0 \le f(\alpha ) \le 1$.

#### Definition 2.3

Let both $Q_{1}$ and $Q_{2}$ be improved scenario fuzzy sets on X. The intersection, union, complement, and inclusion operations on the improved scenario fuzzy set are defined as follows:

1. $$Q_{1} \cap Q_{2} {\text{ = \{ }}(x,\min \{ u_{{Q_{1} }} (x),u_{{Q_{2} }} (x)\} ,\min \{ \eta _{{Q_{1} }} (x),\eta _{{Q_{2} }} (x)\} ,\min \{ v_{{Q_{1} }} (x),v_{{Q_{2} }} (x)\} , \min \{ \theta _{{Q_{1} }} (x),\theta _{{Q_{2} }} (x)\} )|x \in X\} ;$$
2. $$Q_{1} \cap Q_{2} = \{ (x,\max \{ u_{{Q_{1} }} (x),u_{{Q_{2} }} (x)\} ,\max \{ \eta _{{Q_{1} }} (x),\eta _{{Q_{2} }} (x)\} ,\max \{ v_{{Q_{1} }} (x),v_{{Q_{2} }} (x)\} ,\max \{ \theta _{{Q_{1} }} (x),\theta _{{Q_{2} }} (x)\} )|x \in X\}$$
3. $$Q_{1}^{c} { = }\{ (x,u_{{Q_{1} }} (x),\eta_{{Q_{1} }} (x),v_{{Q_{1} }} (x),\theta_{{Q_{1} }} (x))|x \in X\}$$
4. $$Q_{1} \subseteq Q_{2}$$ only when $$u_{{Q_{1} }} (x) \le u_{{Q_{2} }} (x),$$$$\eta_{{Q_{1} }} (x) \le \eta_{{Q_{2} }} (x),$$$$v_{{Q_{1} }} (x) \le v_{{Q_{2} }} (x),\theta_{{Q_{1} }} (x)$$$$\le \theta_{{Q_{2} }} (x)\theta_{{Q_{1} }} (x) \le \theta_{{Q_{2} }} (x)$$.
5. $Q_{1} { = }Q_{2}$ is only $Q_{1} \subset Q_{2}$ and $Q_{1} \supset Q_{2}$.

#### Definition 2.4

Let $X = \{ x_{1} ,x_{2} , \ldots ,x_{n} ,\}$ be a domain of discourse, $Q_{1}$ and $Q_{2}$ are both improved scenario fuzzy sets on $X$. The Hamming distance between $Q_{1}$ and $Q_{2}$ is.

5 $$\begin{aligned} & d(Q_{1} ,Q_{2} ) = \frac{1}{4n}\sum\limits_{i = 1}^{n} {[\left| {u_{{Q_{1} }} (x_{i} ) - u_{{Q_{2} }} (x_{i} )} \right| + \left| {\eta_{{Q_{1} }} (x_{i} ) - \eta_{{Q_{2} }} (x_{i} )} \right| + \left| {v_{{Q_{1} }} (x_{i} ) - v_{{Q_{2} }} (x_{i} )} \right|} \\ & \quad + \left| {\theta_{{Q_{1} }} (x_{i} ) - \theta_{{Q_{2} }} (x_{i} )} \right| + \left| {\rho_{{Q_{1} }} (x_{i} ) - \rho_{{Q_{2} }} (x_{i} )} \right| + \left| {H_{{Q_{1} }} (x_{i} ) - H_{{Q_{2} }} (x_{i} )} \right| + \left| {\frac{{S_{{Q_{1} }} (x_{i} ) - S_{{Q_{2} }} (x_{i} )}}{2}} \right| \\ \end{aligned}$$

Among them, $H_{{Q_{1} }} (x_{i} ),H_{{Q_{2} }} (x_{i} )$ is the exact function of the improved scenario fuzzy sets $Q_{1}$ and $Q_{2}$, and $S_{{Q_{1} }} (x_{i} ),S_{{Q_{2} }} (x_{i} )$ is the scoring function.

#### Definition 2.5

Let $X = \{ x_{1} ,x_{2} , \cdots ,x_{n} \}$ be a domain of discourse, $Q_{1}$ and $Q_{2}$ are both improved scenario fuzzy sets on $X$. $W = \{ \omega_{1} ,\omega_{2} , \cdots ,\omega_{n} \}$ is the weight of the elements in the $Q_{1}$ and $Q_{2}$ sets, and $\omega_{{\text{i}}} \in [0,1](i = 1,2, \cdots ,n),\sum\limits_{i = 1}^{n} {\omega_{i} = 1}$ is the weighted Hamming distance between $Q_{1}$ and $Q_{2}$:

6 $$\begin{aligned} & d_{p} (Q_{1} ,Q_{2} ) = \frac{1}{4n}\sum\limits_{i = 1}^{n} {\omega_{i} \left[ {\left| {u_{{Q_{1} }} (x_{i} ) - u_{{Q_{2} }} (x_{i} )} \right| + \left| {\eta_{{Q_{1} }} (x_{i} ) - \eta_{{Q_{2} }} (x_{i} )} \right| + \left| {v_{{Q_{1} }} (x_{i} ) - v_{{Q_{2} }} (x_{i} )} \right|} \right.} \\ & \quad \left. { + \left| {\theta_{{Q_{1} }} (x_{i} ) - \theta_{{Q_{2} }} (x_{i} )} \right| + \left| {\rho_{{Q_{1} }} (x_{i} ) - \rho_{{Q_{2} }} (x_{i} )} \right| + \left| {H_{{Q_{1} }} (x_{i} ) - H_{{Q_{2} }} (x_{i} )} \right| + \left| {\frac{{S_{{Q_{1} }} (x_{i} ) - S_{{Q_{2} }} (x_{i} )}}{2}} \right|} \right] \\ \end{aligned}$$

## Improving decision-making methods for scenario fuzzy sets

**Fig. 1:**
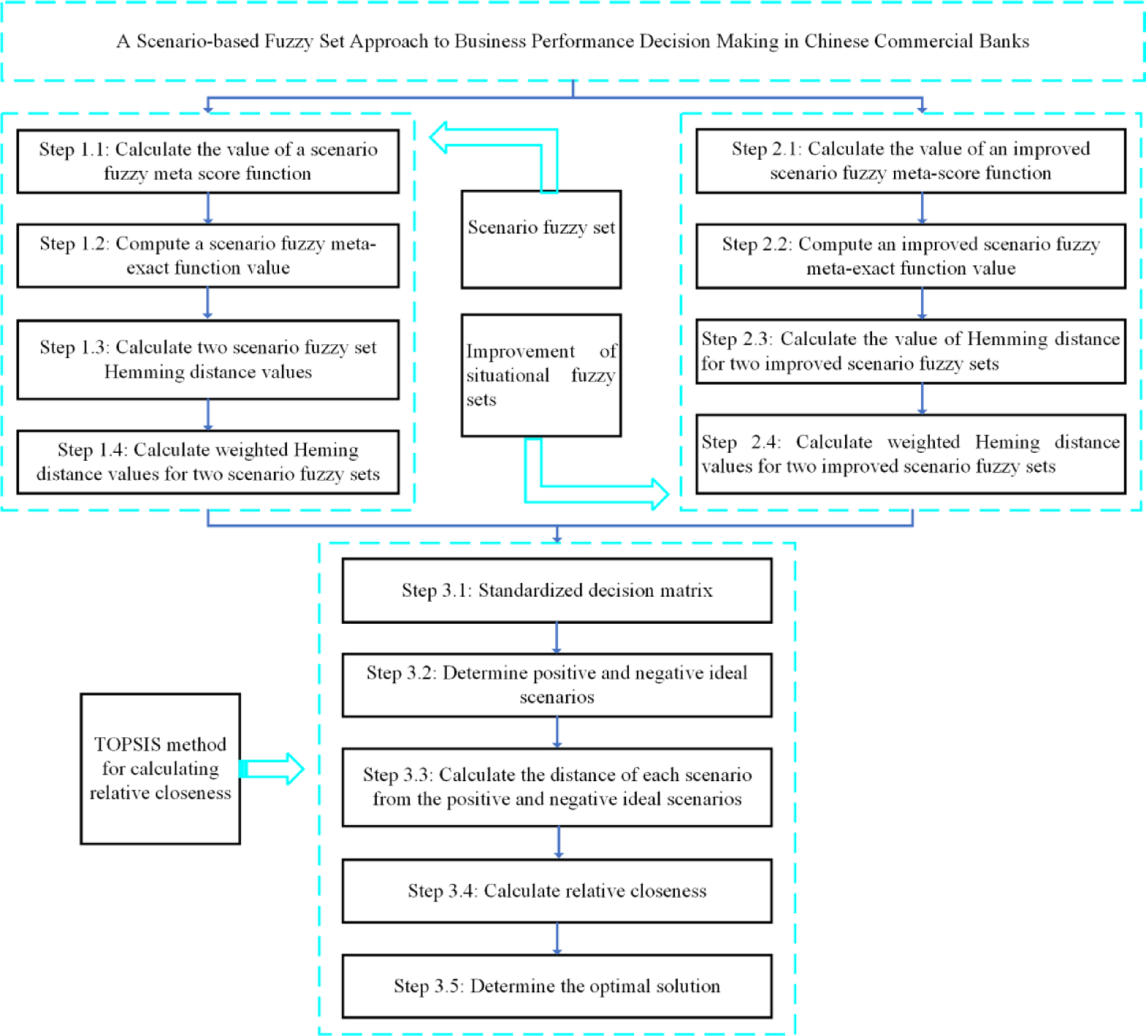
The entire process of calculating bank performance using scenario fuzzy sets.

The operational performance of Chinese commercial banks mainly includes four dimensions: profitability, liquidity, risk, and solvency. In order to better evaluate the rationality of the operational performance of Chinese commercial banks, TOPSIS theory^23^ is introduced. By measuring the Hamming distance with an improved scenario fuzzy set, a multi-attribute decision-making algorithm for an improved scenario fuzzy model is established to select the best solution from alternative options. Multi attribute decision problems are usually expressed using decision matrices, where each element of the matrix represents the value of that attribute. The whole process of the proposed method is shown in Fig. 1.

Assuming $Y = \{ Y_{1} ,Y_{2} , \ldots ,Y_{n} ,\}$ is the solution set, $G = \{ G_{1} ,G_{2} , \ldots ,G_{n} ,\}$ is the attribute set, $W = \{ \omega_{1} ,\omega_{2} , \ldots ,\omega_{n} \}$ is the weight vector $\omega_{{\text{i}}} \in [0,1](i = 1,2, \ldots ,n),\sum\limits_{i = 1}^{n} {\omega_{i} = 1}$ of the attributes.

Decision makers measure all options based on their attributes to obtain an improved scenario fuzzy decision matrix $D = (d_{ij} )_{m \times n}$, where $d_{ij} = (u_{ij} ,\eta_{ij} ,v_{ij} ,\theta_{ij} )$ is the improved scenario fuzzy element representing the improved scenario fuzzy information of option $Y_{i}$ under attribute $G_{j}$. Using the weighted Hamming distance measure on an improved scenario fuzzy set to solve multi-attribute decision-making problems, the specific steps are as follows:

1. Standardization of decision-making information.
  If all attributes are benefit oriented, there is no need to standardize the improved scenario fuzzy decision matrix; If some attributes are cost type attributes, the following method should be used to process the original improved scenario fuzzy decision matrix into a standardized decision matrix $R = (r_{ij} )_{m \times n}$:
  7 $$r_{ij} = \left\{ \begin{gathered} d_{ij} \hfill \\ d_{{_{ij} }}^{c} \hfill \\ \end{gathered} \right.$$
  $$d_{ij}$$ represents $$G_{j}$$ as a benefit attribute, $$d_{{_{ij} }}^{c}$$ represents $$G_{j}$$ as a cost attribute,$$i = 1,2, \ldots ,m;j = 1,2, \ldots ,n$$, $$d_{{_{ij} }}^{c} { = }(u_{ij} ,$$$$\eta_{ij} ,v_{ij} ,\theta_{ij} )^{c} = (v_{ij} ,$$$$\eta_{ij} ,u_{ij} ,\theta_{ij} )$$.
2. Determine the positive ideal scheme $Y^{ + } = \left( {r_{1}^{ + } ,r_{2}^{ + } , \ldots ,r_{n}^{ + } } \right)$ and the negative ideal scheme $Y^{ - } = \left( {r_{1}^{ - } ,r_{2}^{ - } , \ldots ,r_{n}^{ - } } \right)$, where
  8 $$r_{j}^{ + } = (u_{j}^{ + } ,\eta_{j}^{ + } ,v_{j}^{ + } ,\theta_{j}^{ + } ) = \left( {\mathop {\max }\limits_{1 \le i \le m} \{ u_{ij} \} ,\mathop {\min }\limits_{1 \le i \le m} \{ \eta_{ij} \} ,\mathop {\min }\limits_{1 \le i \le m} \{ v_{ij} \} ,\mathop {\min }\limits_{1 \le i \le m} \{ \theta_{ij} \} } \right)$$
  9 $$r_{j}^{ - } = (u_{j}^{ - } ,\eta_{j}^{ - } ,v_{j}^{ - } ,\theta_{j}^{ - } ){ = }\left( {\mathop {\min }\limits_{1 \le i \le m} \{ u_{ij} \} ,\mathop {\max }\limits_{1 \le i \le m} \{ \eta_{ij} \} ,\mathop {\max }\limits_{1 \le i \le m} \{ v_{ij} \} ,\mathop {\max }\limits_{1 \le i \le m} \{ \theta_{ij} \} } \right)$$
3. Calculate the distance measure between $$Y_{i}$$ and $$Y^{ + } ,Y^{ - }$$. Calculate the weighted Hamming distances $$d_{w} (Y_{i} ,Y^{ + } )$$ and $$d_{w} (Y_{i} ,Y^{ - } )$$$$i = 1,2, \ldots ,m.$$ of the $$i$$th scheme $$Y_{i}$$, the ideal scheme $$Y^{ + }$$, and the negative ideal scheme $$Y^{ - }$$ according to the formula.
4. Calculate the relative closeness $C(Y_{i} )$ of scheme $Y_{i}$. Using TOPSIS theory, calculate the positive and negative ideal schemes and attribute weights, sort them according to their values, select the optimal positive and negative ideal schemes, and refer to the formula for relative closeness in the paper:
  10 $$C(Y_{i} ) = \frac{{d_{W} (Y_{i} ,Y^{ - } )}}{{d_{W} (Y_{i} ,Y^{ - } ) + d_{W} (Y_{i} ,Y^{ + } )}},i = 1,2, \ldots ,m.$$
5. Determine the optimal solution. Sort $C(Y_{i} ),(i = 1,2, \ldots ,m)$ according to their value, with higher values indicating better solution $Y_{i}$.

Here, we propose an improved scenario blur method for computing scenario blur sets. The relevant symbols are shown in Table 1.

**Table 1:** Symbol explanation.

Symbol	Meaning	Symbol	Meaning
$X$	Domain	$d_{p} (Q_{1} ,Q_{2} )$	Weighted Hamming distance
$A$	Fuzzy set	$\theta_{A} (x)$	Invalid membership degree
$u_{A} (x)$	Membership degree	$Y$	solution set
$x$	element	$G$	properties
${\text{v}}_{A} (x)$	Non membership degree	$W$	Weight vector
$\pi_{A} (x)$	Hesitation degree	$D$	Scenario fuzzy decision matrix
$\eta_{A} (x)$	Neutral membership degree	$R$	Standardized decision matrix
$\xi_{A} (x)$	Reject affiliation degree	$Y^{ + }$	Ideal solution
$S(\alpha )$	score function	$Y^{ - }$	Negative Ideal Plan
$H(\alpha )$	Exact function	$C(Y_{i} )$	Relative closeness
$d(Q_{1} ,Q_{2} )$	Hamming		

## Case study

To verify the effectiveness of the proposed multi-attribute decision-making method, we used personnel assessment as a case study to validate it.

Example 3.1: In order to clarify the work tasks and responsibilities of employees, fully mobilize their enthusiasm, creativity, and work efficiency, the leaders of a Chinese commercial bank conducted a service quality assessment on six employees of the branch and selected the best annual worker. Assess from four dimensions: work ability, knowledge and skills, service attitude, and service effectiveness. Among them, 6 employees are respectively designated as $Y_{i} (i = 1,2, \ldots ,6)$, and their work ability, knowledge and skills, service attitude, and service effectiveness are represented as $G_{1} ,G_{2} ,G_{3} ,G_{4}$, with their weight vector $W = (0.3,0.18,0.26,0.28)$. Experts gave evaluation scores based on the six staff members’ work ability, knowledge and skills, service attitude, and service effectiveness, including positive $P_{1}$, neutral $P_{2}$, negative $P_{3}$, and ineffective $P_{4}$ (All data generated or analysed during this study are included in this published article), as shown in Table 2.

**Table 2:** Expert rating values for staff.

	$G_{1}$				$G_{2}$				$G_{3}$				$G_{4}$			
	$P_{1}$	$P_{2}$	$P_{3}$	$P_{4}$	$P_{1}$	$P_{2}$	$P_{3}$	$P_{4}$	$P_{1}$	$P_{2}$	$P_{3}$	$P_{4}$	$P_{1}$	$P_{2}$	$P_{3}$	$P_{4}$
$Y_{1}$	0.78	0.05	0.12	0.01	0.75	0.02	0.1	0.13	0.86	0.03	0.07	0.03	0.83	0.02	0.1	0.05
$Y_{2}$	0.75	0.02	0.1	0.03	0.7	0.05	0.2	0.05	0.83	0.04	0.02	0.1	0.79	0.03	0.06	0.09
$Y_{3}$	0.65	0.21	0.08	0.05	0.82	0.01	0.03	0.04	0.84	0.05	0.04	0.05	0.82	0.01	0.05	0.08
$Y_{4}$	0.82	0.01	0.13	0	0.83	0.03	0.08	0.06	0.8	0.03	0.06	0.01	0.8	0.06	0.04	0.07
$Y_{5}$	0.8	0.12	0.05	0.03	0.78	0.07	0.09	0.03	0.85	0.08	0.04	0.02	0.85	0.03	0.05	0.06
$Y_{6}$	0.56	0	0.22	0.08	0.8	0.04	0.01	0.08	0.82	0.03	0.06	0.06	0.86	0.01	0.07	0.04

According to the standardization of decision information in step 1, since the indicators considered for work ability, knowledge and skills, service attitude, and service effectiveness are all benefit based attributes, the original improved scenario fuzzy decision matrix $D$ is its standardized matrix, with $R = D$.

$$R = \left[ {\begin{array}{*{20}c} {0.78} & {0.05} & {0.12} & {0.01} & {0.75} & {0.02} & {0.1} & {0.13} & {0.86} & {0.03} & {0.07} & {0.03} & {0.83} & {0.02} & {0.1} & {0.05} \\ {0.75} & {0.02} & {0.1} & {0.03} & {0.7} & {0.05} & {0.2} & {0.05} & {0.83} & {0.04} & {0.02} & {0.1} & {0.79} & {0.03} & {0.06} & {0.09} \\ {0.65} & {0.21} & {0.08} & {0.05} & {0.82} & {0.01} & {0.03} & {0.04} & {0.84} & {0.05} & {0.04} & {0.05} & {0.82} & {0.01} & {0.05} & {0.08} \\ {0.82} & {0.01} & {0.13} & 0 & {0.83} & {0.03} & {0.08} & {0.06} & {0.8} & {0.03} & {0.06} & {0.01} & {0.8} & {0.06} & {0.04} & {0.07} \\ {0.8} & {0.12} & {0.03} & {0.05} & {0.78} & { \, 0.07} & {0.09} & {0.03} & {0.85} & {0.08} & {0.04} & {0.02} & {0.85} & {0.03} & {0.05} & {0.06} \\ {0.56} & 0 & { \, 0.22} & {0.08} & {0.8} & {0.04} & {0.01} & {0.08} & {0.82} & {0.03 \, } & {0.06} & {0.06} & {0.86} & {0.01} & {0.07} & {0.04} \\ \end{array} } \right]$$ Determine the positive and negative ideal $Y^{ + } ,Y^{ - }$ according to step 2

$$Y^{ + } {\text{ = }}\left\{ {{\text{(}}0.82,0,0.05,0),(0.83,0.01,0.01,0.03),(0.86,0.03,0.02,0.01),(0.86,0.01,0.04,0.04)} \right\}$$

$$Y^{ + } {\text{ = }}\left\{ {{\text{(}}0.56,0.21,0.22,0.08),(0.7,0.07,0.2,0.13),(0.8,0.08,0.07,0.1),(0.79,0.06,0.1,0.09)} \right\}$$

Calculate the weighted Hamming distances $d_{w} (Y_{i} ,Y^{ + } )$ and $d_{w} (Y_{i} ,Y^{ - } )$ according to step 3, as shown in Table 3.

**Table 3:** _Positive and negative ideal hamming distance_.

Positive ideal weighted hamming distance	Price	Negative ideal weighted hamming distance	Price
$d_{w} (Y_{1} ,Y^{ + } )$	0.0992	$d_{w} (Y_{1} ,Y^{ - } )$	0.1006
$d_{w} (Y_{2} ,Y^{ + } )$	0.0956	$d_{w} (Y_{2} ,Y^{ - } )$	0.0958
$d_{w} (Y_{3} ,Y^{ + } )$	0.0981	$d_{w} (Y_{3} ,Y^{ - } )$	0.0984
$d_{w} (Y_{4} ,Y^{ + } )$	0.0966	$d_{w} (Y_{4} ,Y^{ - } )$	0.1025
$d_{w} (Y_{5} ,Y^{ + } )$	0.1016	$d_{w} (Y_{5} ,Y^{ - } )$	0.1049
$d_{w} (Y_{6} ,Y^{ + } )$	0.0932	$d_{w} (Y_{6} ,Y^{ - } )$	0.0928

Calculate the relative closeness $C(Y_{i} )$ according to step 4, as shown in Table 4.

**Table 4:** Relative closeness and ranking of improved scenario fuzzy sets.

Relative closeness	Relative closeness value	Ranking
$C(Y_{1} )$	0.5036	3
$C(Y_{2} )$	0.5004	5
$C(Y_{3} )$	0.5006	4
$C(Y_{4} )$	0.5147	1
$C(Y_{5} )$	0.5081	2
$C(Y_{6} )$	0.4990	6

Determine the optimal solution according to step 5. Sort $C(Y_{i} )(i = 1,2, \ldots ,m)$ according to their value, with higher values indicating better solution $Y_{i}$. From Table 3, it can be seen that $C(Y_{4} ) > C(Y_{5} ) > C(Y_{1} ) > C(Y_{3} ) > C(Y_{2} ) > C(Y_{6} )$, also known as $Y_{4} \succ Y_{5} \succ Y_{1} \succ Y_{3} \succ Y_{2} \succ Y_{6}$, is the optimal $Y_{4}$ scheme due to the higher $Y_{4}$ value.

## Comparative analysis of situational fuzzy sets and improved situational fuzzy sets

This article introduces the scenario fuzzy set and the improved scenario fuzzy set, compares and analyzes the two, and introduces an invalid dimension to the improved scenario fuzzy set, which has made slight changes to the performance evaluation of Chinese commercial banks, as shown in Table 5.

**Table 5:** Relative closeness and ranking between scenario fuzzy set and improved scenario fuzzy set.

Relative closeness	PFS	Ranking	ISFS	Ranking
$C(Y_{1} )$	0.6397	3	0.5036	3
$C(Y_{2} )$	0.5221	6	0.5004	5
$C(Y_{3} )$	0.5956	4	0.5006	4
$C(Y_{4} )$	0.7132	1	0.5147	1
$C(Y_{5} )$	0.6691	2	0.5081	2
$C(Y_{6} )$	0.5588	5	0.4990	6

According to Table 5, the relative closeness ranking of the Scenario Fuzzy Set (PFS) is $C(Y_{4} ) > C(Y_{5} ) > C(Y_{1} ) > C(Y_{3} ) > C(Y_{6} ) > C(Y_{2} )$, which is the excellent employee ranking scheme $Y_{4} \succ Y_{5} \succ Y_{1} \succ Y_{3} \succ Y_{6} \succ Y_{2}$; The relative closeness ranking of the Improved Scenario Fuzzy Set (ISFS) is as follows: $C(Y_{4} ) > C(Y_{5} ) > C(Y_{1} ) > C(Y_{3} ) > C(Y_{2} ) > C(Y_{6} )$ stands for Excellent Employee Ranking Scheme: $Y_{4} \succ Y_{5} \succ Y_{1} \succ Y_{3} \succ Y_{2} \succ Y_{6}$, The scenario fuzzy set and the improved scenario fuzzy set scheme are basically the same, but there are differences in the ranking of employee $Y_{2}$ and employee $Y_{6}$. The scores of various indicators in the $Y_{6}$ scenario fuzzy set for the main employee are (0.56, 0, 0.22, 0.08), with a Positive score of 0.56, which is the lowest among all positive indicators, and a negativity score of 0.22, which is the highest among all negative indicators. The positive indicators for employee $Y_{2}$ are all greater than 0.7, while the negative indicators are less than 0.22. This result is consistent with the bank’s performance and better reflects the overall interests of the bank. The evaluation results also appear more objective.

## Management recommendations

Based on the above bank performance evaluation methods, the following management recommendations are proposed with the aim of optimizing the performance management system and improving the overall operation level.1. Clarify the performance evaluation indicators and strengthen the construction of the indicator system. Bank management should clarify the key performance indicators and utilize the improved situational fuzzy set model to subdivide the indicators into positive, neutral, negative and ineffective categories, so as to achieve multi-dimensional and detailed performance evaluation and provide a more comprehensive basis for decision-making.2. Adopt the fuzzy data processing technology to enhance the accuracy of evaluation. By optimizing the fuzzy attributes and reducing the uncertainty and ambiguity in the data, it ensures that the performance evaluation results are closer to the actual situation and enhances the management’s ability to judge the overall operation status of the bank.3. Using Hamming distance to conduct difference analysis and guide special improvement. Using Hamming distance to measure the relationship and differences between different indicators, identify the key factors affecting the bank’s performance, and provide a quantitative basis for formulating targeted improvement measures.4. Introducing TOPSIS multi-attribute decision-making method to realize scientific preference. Combined with the fuzzy evaluation results, the TOPSIS method is used to help banks prioritize the selection and implementation of performance improvement measures to ensure the scientific and effective strategies.5. Establish a continuous evaluation and feedback mechanism. Regularly examine the adaptability of the performance evaluation model and indicator system, and dynamically adjust the evaluation model in conjunction with changes in the actual operating environment to ensure the continued effectiveness of performance management.6. Strengthen training and capacity building. To ensure the correct application and understanding of the model, systematic training should be provided to managers and employees to popularize the basic principles and operation methods of fuzzy set, Hamming distance and TOPSIS in order to enhance the overall performance management level. By implementing the above recommendations, the bank will build a more scientific, flexible and comprehensive performance management system, which will significantly improve management efficiency and market competitiveness.

## Conclusion

In this paper, we discuss service quality as an important factor affecting the operational efficiency of commercial banks and propose an evaluation method based on an improved scenario fuzzy set model. Our study finds that the level of service quality directly affects the long-term development prospects of banks, and therefore, the establishment of an effective evaluation system is crucial for improving service quality. In our model, by introducing the contribution of invalid affiliation and weighted Hemming distance, we are able to reflect the service quality of bank staff more accurately. The results of the study not only verify the validity of the model, but also reveal the following key points:1. Importance of comprehensive satisfaction: the assessed comprehensive satisfaction indicators provide bank managers with intuitive feedback, helping them to identify service quality deficiencies and formulate improvement measures.2. Superiority of multi-attribute decision-making model: the decision-making model based on the TOPSIS methodology shows good potential for application for the commercial banks to provide more scientific support in the fierce market competition.3. Directions for future research: it is recommended to further explore the dynamic service quality assessment method that combines customer feedback and real-time data to adapt to the rapidly changing market environment.

Overall, the model proposed in this paper provides solid data support for commercial banks to improve their service quality, advances the development of service quality evaluation methods, and will provide a deeper theoretical foundation and reference for practical application in the future.

## Data Availability

All data generated or analysed during this study are included in this published article.
